# Reforming CO_2_ bio-mitigation utilizing *Bacillus cereus* from hypersaline realms in pilot-scale bubble column bioreactor

**DOI:** 10.1038/s41598-024-56965-8

**Published:** 2024-03-16

**Authors:** Rachael J. Barla, Smita Raghuvanshi, Suresh Gupta

**Affiliations:** Faculty Division-1, Department of Chemical Engineering, Birla Institute of Technology and Science (BITS), BITS PILANI, Pilani, 333031 Rajasthan India

**Keywords:** Biotechnology, Engineering

## Abstract

The bubble column reactor of 10 and 20 L capacity was designed to bio-mitigate 10% CO_2_ (g) with 90% air utilizing thermophilic bacteria (*Bacillus cereus* SSLMC2). The maximum biomass yield during the growth phase was obtained as 9.14 and 10.78 g L^−1^ for 10 and 20 L capacity, respectively. The maximum removal efficiency for CO_2_ (g) was obtained as 56% and 85% for the 10 and 20 L reactors, respectively. The FT-IR and GC–MS examination of the extracellular and intracellular samples identified value-added products such as carboxylic acid, fatty alcohols, and hydrocarbons produced during the process. The total carbon balance for CO_2_ utilization in different forms confirmed that *B. cereus* SSLMC2 utilized 1646.54 g C in 10 L and 1587 g of C in 20 L reactor out of 1696.13 g of total carbon feed. The techno-economic assessment established that the capital investment required was $286.21 and $289.08 per reactor run of 11 days and $0.167 and $0.187 per gram of carbon treated for 10 and 20 L reactors, respectively. The possible mechanism pathways for bio-mitigating CO_2_ (g) by *B. cereus* SSLMC2 were also presented utilizing the energy reactions. Hence, the work presents the novelty of utilizing thermophilic bacteria and a bubble column bioreactor for CO_2_ (g) bio-mitigation.

## Introduction

The carbon dioxide (CO_2_) concentration in the atmosphere has increased by 31% from 1958 to 2022, reaching 417 parts per million in December 2022. This change indicates that CO_2_ concentrations have significantly increased, and by 2050, the atmospheric CO_2_ concentration will exceed 550 ppm if no action is taken to reduce emissions^1^. To keep global warming below 1.5 °C, all countries must achieve net-zero CO_2_ emissions by 2050 while reducing emissions of other GHG and non-CO_2_ contributors^2^. In response to rising CO_2_ emissions, governments of many countries have imposed strict limits on the emission standards of facilities like power plants, cement factories, petroleum refineries, etc. The Air (Prevention and Control of Pollution) Act, 1981, and Environment (Protection) Act, 1986, established an industrial emission standard of 400 mg m^−3^ for CO_2_ in India. The European Clean Air Act (42 U.S.C. 7401 et seq.) protects human and environmental health from air pollution. It requires EPA to set minimum national air quality standards and gives states primary responsibility for compliance. An emission standard of 450 mg m^−3^ for CO_2_ has been set in the country. The federal Clean Air Act (CAA) (42 U.S.C. 7401 et seq.) regulates all air emissions with 350 mg m^−3^ of CO_2_. EPA established National Ambient Air Quality Standards (NAAQS) to protect public health and the environment under the 1970 CAA. Article 1 of Japan regulates the emission and dispersal of soot, smoke, volatile organic compounds, and particulates from factories and businesses with a CO_2_ emission standard of 220 mg m^−3^ to protect public health and the environment. The Chinese Air Pollution Control Act regulates air pollution from coal, industry, motor vehicles and vessels, dust, and agriculture. It addresses air pollutants and greenhouse gases like aerosol particles, sulphur dioxide, nitrogen oxides, volatile organic compounds, ammonia, and CO_2_ with an emission standard of 400 mg m^−3^.

Following the regulations applied by different countries, the bio-mitigation process meets the goal of reducing air pollution and the Sustainable Development Goals (SDGs), such as establishing good health and well-being, growing affordable and clean energy, increasing industry, innovation, and infrastructure, and organizing climate action. According to the International Energy Agency (IEA), cement production accounted for approximately 7% of global CO_2_ emissions in 2020. This makes it one of the largest industrial sources of GHG emissions^3^. On average, the cement industry emits around 0.7–0.8 metric tons of CO_2_ per ton of cement produced, with a high CO_2_ concentration in their flue gases of about 10–30%^4^. Therefore, it is necessary to develop technologies to mitigate CO_2_ from the emission sources.

CO_2_ can be mitigated from the emission sources by traditional methods such as adsorption^5^, absorption^6^, and membrane separation^7^. The bio-mitigation process is a cutting-edge method that can potentially eliminate a few drawbacks of traditional CO_2_ mitigation methods. This method uses microorganisms like bacteria or algae^8^. According to earlier reports, much research has been done on microalgae for CO_2_ bio-mitigation. However, there are limitations to microalgae-based mitigation measures, such as the requirement for large amounts of water and area for growing ponds, enough light intensity, and a costly biomass harvesting process^9–11^. As an alternative, the potential to utilize bacteria to lower CO_2_ emissions has vast untapped potential^12^. Bacteria may use less water and space than microalgae because they can grow easily in any reactor without a light source. Different bacterial species require different substrates, including carbon, nitrogen, sulphate, and phosphorus, for optimum growth. Various kinds of bacteria, such as *Pseudomonas*^13^, *Bacillus cereus*^14^, *Serratia* sp. ISTD04^15^, *Bacillus* sp. ISTS2^16^ has been used for CO_2_ bio-mitigation.

The bacteria facilitate the utilization of CO_2_ from the gas mixture as substrates; this reduces the quantity of CO_2_ released to the environment and thus reduces its adverse impact. As gas concentrations increase, the production of fatty alcohols increases at the expense of proteins and carbohydrates in the cells^17^. A thermophilic bacteria has shown a higher removal efficiency than mesophilic bacteria^18^. Thermophilic bacteria can be obtained from harsh and scorching environments like salt lakes and waste landfills. Due to their greater heat tolerance, thermophilic bacteria isolated from harsh environments can mitigate CO_2_ effectively from emission sources^19^. The bio-mitigation process uses thermophilic bacteria to convert the CO_2_ into other usable forms; however, adding foreign compounds as solvents is required in other physical and chemical processes, requiring additional separation and disposal. CO_2_ is absorbed and used by bacteria as an excellent carbon source and a valuable feedstock for producing industrial chemicals and fuels. It offers a practical answer to the paradox between rising energy use and falling CO_2_ emissions^20^.

CO_2_ mitigation by bacterial species was carried out in various reactors with varying volumes, such as batch reactors (Erlenmeyer flasks) (800 mL)^21^, airlift reactor (5.6 L)^22^, and continuous stirred tank reactors (CSTR) (3 L)^23^. The success of the CO_2_ bio-mitigation process depends on the higher cell concentration. The reactions in an Erlenmeyer flask are of low volume, while a greater volume is required to get the desired reduction and conversion of products^24^. The packed bed columns have drawbacks such as gas channeling, temperature inconsistency, and difficulty in replacing packing materials and cleaning, limiting the use of packed-bed biofilters in large-scale operations^25^. Airlift bioreactor lacks mixing at high-pressure drops and is unsuitable for high cell concentration^26^. The major drawbacks of CSTR are the high power consumption and the possibility of damaging the shear-sensitive cells at higher agitation speeds^27^. The power consumption is higher in CSTR due to more mechanical parts, while packing materials make the packed bed reactor expensive during scale-up^28,29^.

A bubble column bioreactor can overcome the demerits of the reactors mentioned above for the bio-mitigation of CO_2_. It outperforms substitute reactors in terms of both design and operation. Bubble column reactor does not need packing materials and an agitator, unlike packed bed reactor and CSTR, respectively. Additionally, it is suitable to produce and operate at higher cell concentrations and requires less time for cleaning and disinfection. Due to the absence of internal parts in the bubble column bioreactor, there is less probability of media contamination during operation. Its high mass transfer efficiency helps reduce CO_2_ emissions and improve product quality^30,31^. As fewer moving parts and less energy are needed to run the bubble column reactor, it has a lower operating cost. The design of a bubble column reactor facilitates proper mixing, gas distribution, heat, and mass transfer, enabling higher bacterial biomass productivity, which makes it feasible to scale up to the industrial level^31^.

This research designed and fabricated a pilot-scale bubble column bio-reactor with a working volume of 10 and 20 L. In semi-continuous modes, the reactor was run as a sequence of a 10 L single-column reactor and a 20 L double-column reactor in series. The bio-mitigation experiment of the gas mixture of 10% CO_2_ (g) and 90% air was carried out separately in 10 L and 20 L working volumes with the mixed thermophilic bacteria obtained from Sambhar Salt Lake (SSL), Rajasthan. Multiple parametric values such as pH, dissolved oxygen (DO), salinity, total organic carbon (TOC), and total inorganic carbon (TIC) were estimated to analyze the effect of biomass growth on CO_2_ mitigation efficiency. Fourier Transform—Infrared Spectroscopy (FT-IR) and Gas Chromatography-Mass Spectroscopy (GC–MS) analyses were also used to assess the product formation during CO_2_ mitigation. The techno-economic analysis finally demonstrated the effectiveness of the bioreactor's working modes. Based on the energy reactions, a suggested metabolism pathway for bio-mitigating CO_2_ (g) by the bacteria is also presented.

## Materials and methods

### Bacterial selection, culture conditions, and inoculation

The thermophilic bacteria used in the study were isolated from sludge and water samples collected from the hypersaline environment of Sambhar Salt Lake in the Indian state of Rajasthan. The probability of finding the carbon-utilizing bacteria, denitrificans, and desulphuricans in such environments is higher and is suitable for the CO_2_ bio-mitigation process^32,33^. The samples brought to the lab were stored at 4 °C in a sterile condition to avoid external contamination.

All the solutions used in the experiment were prepared using deionized (DI) water and sterilized by autoclaving. The selective enrichment culture technique was applied to the sludge and water sample to enrich the mixed bacterial culture. In a 500 mL Erlenmeyer flask, the culture was incubated with 10 mM sodium bicarbonate (NaHCO_3_) as the carbon source in an orbital shaker (Metrex Scientific, India) for 48 h at 120 rpm and 37 °C. The grown culture was further enriched by continuously purging CO_2_ (g) as the carbon source for 6 days. Later, this grown culture was enriched using nutrient broth containing sodium chloride (5 g L^−1^), peptone (5 g L^−1^), and beef extract (1.5 g L^−1^) to get inoculum, which was used for mitigation studies. The inoculum was utilized at a volume of 10% of the total working volume of the reactor (1 L: 10 L and 2 L: 20 L). The MSM media fed in the reactor for the cultivation of bacteria consisted of the following salts (g L^−1^): Potassium Nitrate (KNO_3_)—1, Dipotassium hydrogen phosphate (K_2_HPO_4_)—1, Ammonium Chloride (NH_4_Cl)—0.16, Sodium Thiosulphate (Na_2_S_2_O_3_)—24.5, Sodium Chloride (NaCl)—35. The quantity of minimal salt media (MSM) prepared depended on the reactor’s working volume (10 L and 20 L). The pH of the freshly prepared media was around 9.6, and the salinity was 48 ppm.

### Bubble column bioreactor

**Figure 1:**
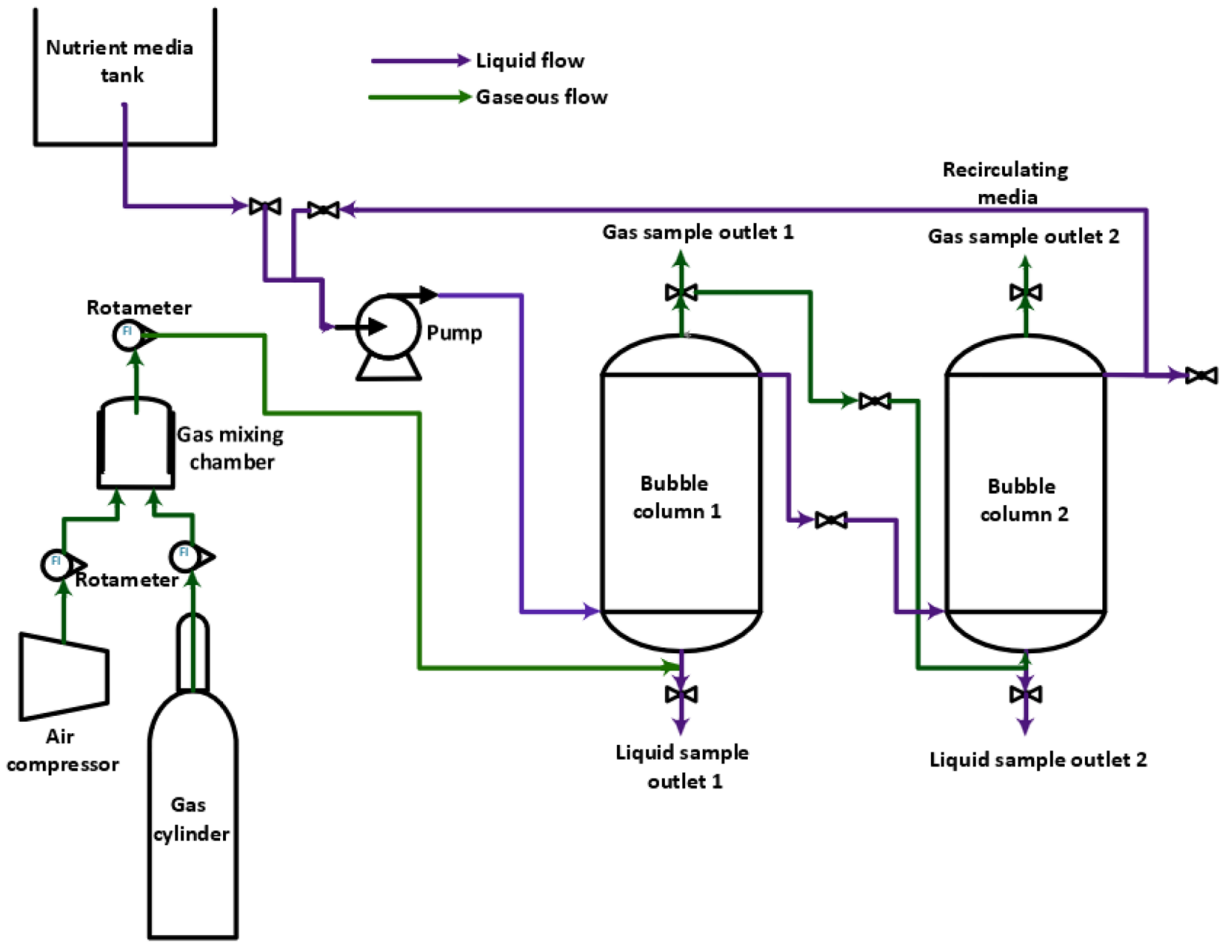
The schematic of the semi-continuous bubble column bioreactor setup.

The bubble column bio-reactor system used for the bio-mitigation study is represented in Fig. 1. The bio-reactor configuration comprised a 10 L volume of two glass bubble columns with 2 L of head space per column. The reactor could be operated with either a single column with a volume of 10 L or both columns in series with a volume of 20 L. The essential design parameters of a bubble column reactor are the column's height and diameter and the sparger opening diameter. The reactor was designed with each column having a height and diameter of 24 and 6.5 inches, respectively. A ring sparger was installed at the bottom of each column with a sparger opening diameter of 0.8 mm and a triangular pitch. A perforated stainless steel 304 mesh sieve plate was placed just above it. The gas was sparged through the small holes that distributed the air as fine bubbles in the bioreactor to facilitate adequate aeration and mass transfer of CO_2_ from the gas to the liquid phase^31^. The 10 L MSM and circulating nutrient media tanks were located in the system’s rear. A peristaltic pump pumped the medium through the pipes. The gas mixture cylinder was connected to the column via an air filter and rotameter. The media and gas mixture was fed into the column from its base. The bacterial growth and CO_2_ abatement occurred inside the bubble column reactor.

Before the start of the experiment, the columns were meticulously steam-cleaned and sanitized with sodium hypochlorite solution to prevent contamination. The media was continuously transferred from the first column to the second column, and the gas collected in the headspace of the first column was fed from the bottom of the second column. At the top of both columns was a gas sampling port, through which gas samples were measured with a flue gas analyzer. A peristaltic pump was attached to the second column to remove the liquid sample for further analysis. The bottom sieve plate of the column ensured proper gas distribution and prevented biomass from collecting on the sparger and clogging the holes.

## Experimental procedures

### Inoculation and experimental condition on the initial day

The inoculation was accomplished by adding the inoculum (1.322 at (optical density) OD_600nm_) into the reactor. 10% CO_2_ (g) from a pressurized cylinder and 90% moisture-free air (g) from an air compressor were mixed in the mixing chamber. The reactor in this study operated in a semi-continuous mode where the medium (MSM) was fed at the beginning of the reactor, and CO_2_ (g) gaseous mixture was continuously fed from the mixing chamber to the column’s inlet at the volumetric flow rate of 2 L min^−1^. The experimental run was continuously performed for 11 days (264 h). The column was sealed entirely and made leakproof for gaseous and liquid phases before starting the experiment. The abiotic test with the same conditions was performed with only air (g) feed from the compressor to check for bacteria growth in the reactor without CO_2_ (g).

The experiments were performed separately for the bioreactor’s 10 L and 20 L working volumes by utilizing a single column and both columns in series. The parameters measured at the reactor’s start-up were the following: (1) initial OD of the media in the column (0.052); (2) initial dry-weight biomass (0.92 g L^−1^); (3) dissolved CO_2_ (0.03 g L^−1^); (4) dissolved O_2_ (0.01 g L^−1^); (5) pH (8.6); (6) salinity (6.20 ppt); (7) headspace CO_2_ and O_2_ concentration (0.02% and 20.90%); and (8) temperature (32 °C). These measurements were utilized as the baseline information for the daily parametric measurements. The other parameters measured for various calculations were carbonate ions, bicarbonate ions, TOC, and colony formation unit (CFU). The parameters were measured after every 12 h for the entire bioreactor operation. The samples were collected and analyzed in triplicates, and the standard deviation represented the variation between the datasets.

### Analytical procedures

A flue gas analyzer (FGA) (Indus Scientific, India) was used to monitor the CO_2_ (g) concentration directly at the bioreactor's entrance and outflow. A culture media sample of 10 mL was used for the OD measurement using a UV–VIS Spectrophotometer (Evolution 201, Thermo Scientific, USA), considering the prepared MSM media as the reference. The sample’s pH, DO, and salinity were measured directly with the probes using the pH meter (Eco Testr, Eutech Instruments, Singapore) and water and soil analysis kit (Khera Instruments, India), respectively. The modified Walkley and Black method was used to calculate the TOC of the biomass^34^. 1 mL sample of suspended biomass was reacted with 1 mL of potassium dichromate (K_2_Cr_2_O_7_) solution (1 N) and 2 mL of 98% concentrated sulphuric acid (H_2_SO_4_). The OD of the final solution was measured at 649 nm to obtain the TOC values.

A 20 mL culture media was filtered using Whatman 0.22 µm cellulose membrane filter paper to determine dissolved CO_2_ concentration in filtrate using a dissolved CO_2_ analyzer (OxyGuard, Pentair, USA). The above-filtered filter paper was air-dried in an oven at 60 °C, and the dry-weight biomass was estimated by comparing the weighed filter paper before and after drying. Standard procedures were utilized to determine the carbonate (CO_3_^2-^) and bicarbonate (HCO_3_^-^) concentration in the above filtrate by titrating it against 0.2 N sulphuric acid (H_2_SO_4_) using phenolphthalein and methyl orange indicators^35^.

The live bacterial species in the reactor involved in the CO_2_ bio-mitigation process from both reactor runs were plated daily to monitor the growth of the bacteria. The species were plated from the first column of the 10 L reactor and the second column of the 20 L reactor. The final sample plated on the last day (11th day) of the reactor run was isolated and sent for further identification by 16 s rRNA sequencing to Anuvanshiki (OPC) Pvt. Ltd., Delhi, India. The bacteria DNA was used in polymerase chain reaction (PCR) to amplify 16 s 27F forward and 1492R reverse primers. Only the forward primer sequence (GAAGGNACCGCATAAGACTTG) was further utilized in the sequencing. The amplicon was gel-eluted, and the sample was sequenced by Sanger’s method of DNA sequencing. The sequencing results were assembled and compared with the National Center for Biotechnology Information (NCBI) database.

### Bacterial growth rate and CO_2_ mitigation efficiency

The biomass growth rate is the rate at which cell mass or number increases as a function of time while utilizing a substrate. With time, the increase in the turbidity of aqueous media indicated the growth of the bacterial cells, which could be determined by measuring the dry-weight biomass. The biomass productivity (*P*_max_, g L^−1^ h^−1^) and the specific growth rate (*µ*, hour^−1^), were derived using Eqs. (1 and 2).

1 $$P_{max} = \frac{{\left( {X_{t} - X_{o} } \right)}}{{\left( {t_{1} - t_{o} } \right)}}$$

2 $$\mu = \frac{{\ln X_{t} - \ln X_{o} }}{{t_{1} - t_{o} }}$$

where *X*_o_ and *X*_t_ are the initial (*t*_o_) and final day (*t*_1_) biomass concentration (g L^−1^).

The removal efficiency (*RE*, %), inlet loading (*IL*, g L^−1^ h^−1^), elimination capacity (*EC*, g L^−1^ h^−1^), and residence time (*RT*, min) were calculated using Eqs. (3–6), resctively, to assess the performance of the bubble column reactor for CO_2_ bio-mitigation. The system's elimination capacity is considered one of the bioreactor's most critical operational parameters as it defines its performance and design criterion.

3 $$RE = \frac{{C_{in} - C_{out} }}{{C_{out} }} \times 100\%$$

4 $$EC = \frac{{Q \times \left( {C_{in} - C_{out} } \right)}}{V}$$

5 $$IL = \frac{{Q \times C_{in} }}{V}$$

6 $$RT = \frac{V}{Q}$$

where *C*_in_ and *C*_out_ are the inlet and outlet CO_2_ (g) concentrations, *Q* is the inlet gas flow rate (L min^−1^), and *V* is the working volume of the reactor (L).

Further, the CO_2_ (g) biofixation rate $\left( {R_{{CO_{2} }} } \right)$ was calculated by Eq. (7).

7 $$R_{{CO_{2} }} = C_{c} \times P_{max} \times \frac{{M_{{CO_{2} }} }}{{M_{c} }}$$

where $M_{{CO_{2} }}$ is the molecular weight of CO_2,_ *M*_c_ is the atomic weight of carbon, and *C*_c_ is the carbon content in the biomass obtained through the TOC calculation.

### FT-IR and GC–MS analysis of the biomass

The biomass obtained at the end of the experimental run of 264 h was utilized to evaluate the formation of metabolites or primary products formed during the CO_2_ bio-mitigation using FT-IR (Frontier, Perkin Elmer, USA) and GC–MS (QP-2010 Plus, Shimadzu, Japan) analysis. The aqueous media was centrifuged at 10,000 rpm at 4 °C for 20 min for FT-IR and GC–MS analysis. Then, the obtained biomass and supernatant were freeze-dried in a lyophilizer (ScanVac freeze-dryer, LaboGene, Scandinavia). The acquired lyophilized samples were pelletized and analyzed using FT-IR. Freeze-dried biomass and potassium bromide (KBr) were mixed in a ratio of 1:10 to create the pellets. Following the protocol described in the literature, the FT-IR was performed over a range of 300–4000 cm^−1^ with a resolution of 4 cm^−1^^36^.

For GC–MS analysis, two forms of samples were obtained after centrifugation: the IC (cell lysate) and the EC (cell-free supernatant). The acquired cell lysate was treated using a cell lysis buffer (50 mM Tris–HCl, pH—7.5, 1 mM phenyl methane sulfonyl fluoride (PMSF), and 0.2% lysozyme). The cells were lyzed by sonication at 15,000 Hz at 4 °C for 5 min at 5 s intervals. The lysate was centrifuged at 10,000 rpm at 4 °C for 20 min. The cell debris was discarded, and the obtained supernatant was utilized for solvent extraction. Extraction was carried out using a combination of chloroform (CHCl_3_) and methanol (CH_3_OH) with a purity of greater than 99.6% in a ratio of 2:1. To facilitate phase separation, the supernatant, and solvent were combined and reacted in an incubator orbital shaker for 2 h at 120 rpm and 37 °C. The aqueous phase obtained after separation was discarded, and the organic phase was used further for the GC–MS analysis. The identical solvent extraction method was applied to the previously collected cell-free supernatant for the GC–MS analysis.

A polytetrafluoroethylene (PTFE) syringe filter with a 25 mm diameter and a pore size of 0.22 µm was used to filter the final intra- and extracellular samples. The samples were finally analyzed using a DB-5 MS column in the GC–MS system with Helium as the carrier gas. A 280 °C GC–MS interface temperature was used to inject the 1 µL concentrated sample into the system. The detailed procedure followed for extracting the fatty alcohol acids is described in the literature^36^. The data was compared to the mass spectrum library already included in the GC–MS instrument (NIST-05 and Wiley-8).

## Results and discussion

The results obtained from the semi-continuous studies of the bio-mitigation of CO_2_ (g) in 10 L and 20 L reactors are discussed in the succeeding sections. All the obtained data are statistically analyzed with the OriginPro 8.5 software.

### Bacterial identification

**Figure 2:**
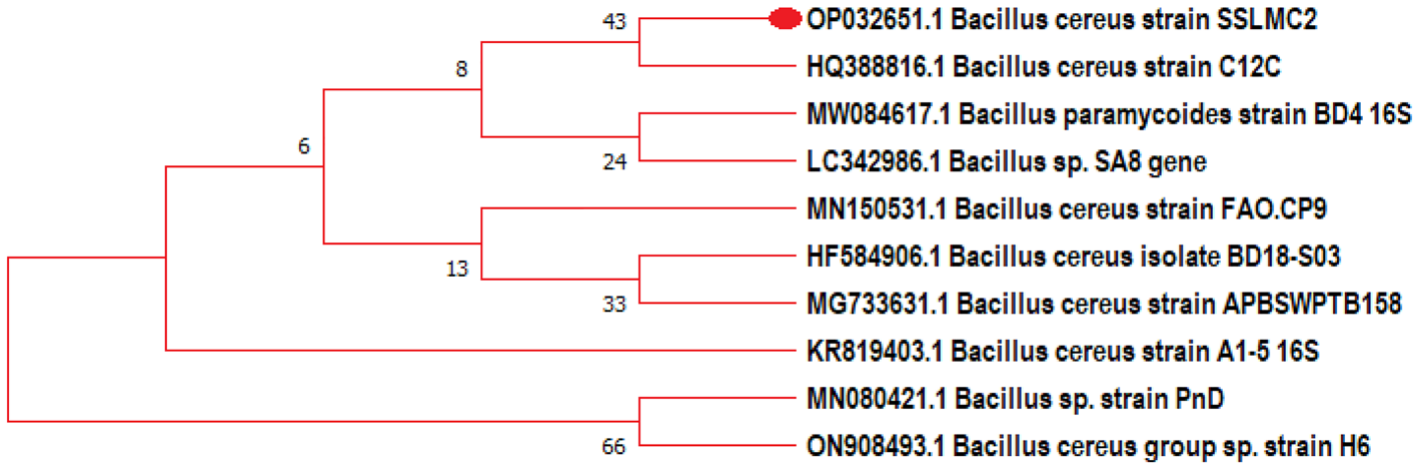
The phylogenetic tree depicting the relationship of the representative sequence of the taxonomic unit.

The plated sample with a single colony of bacteria was sent for identification. Gene sequencing data were used to construct a phylogenetic tree (Fig. 2) to depict the diverse bacterial taxonomy. According to the phylogenetic and molecular analyses, the sample has a nucleotide-level homology of 99.12% with *Bacillus cereus* strain SSLMC2. The gene sequencing analysis showed that the bacteria responsible for reducing CO_2_ in the system belongs to the *Bacillus* class. Based on the percentage identity and E-Value in the blast results, it represents the probability of the alignment occurring for a specific sequence, i.e., a value close to zero means that practically no related sequence was present in the sample.

### CO_2_ (g) bio-mitigation and biomass production

**Figure 3:**
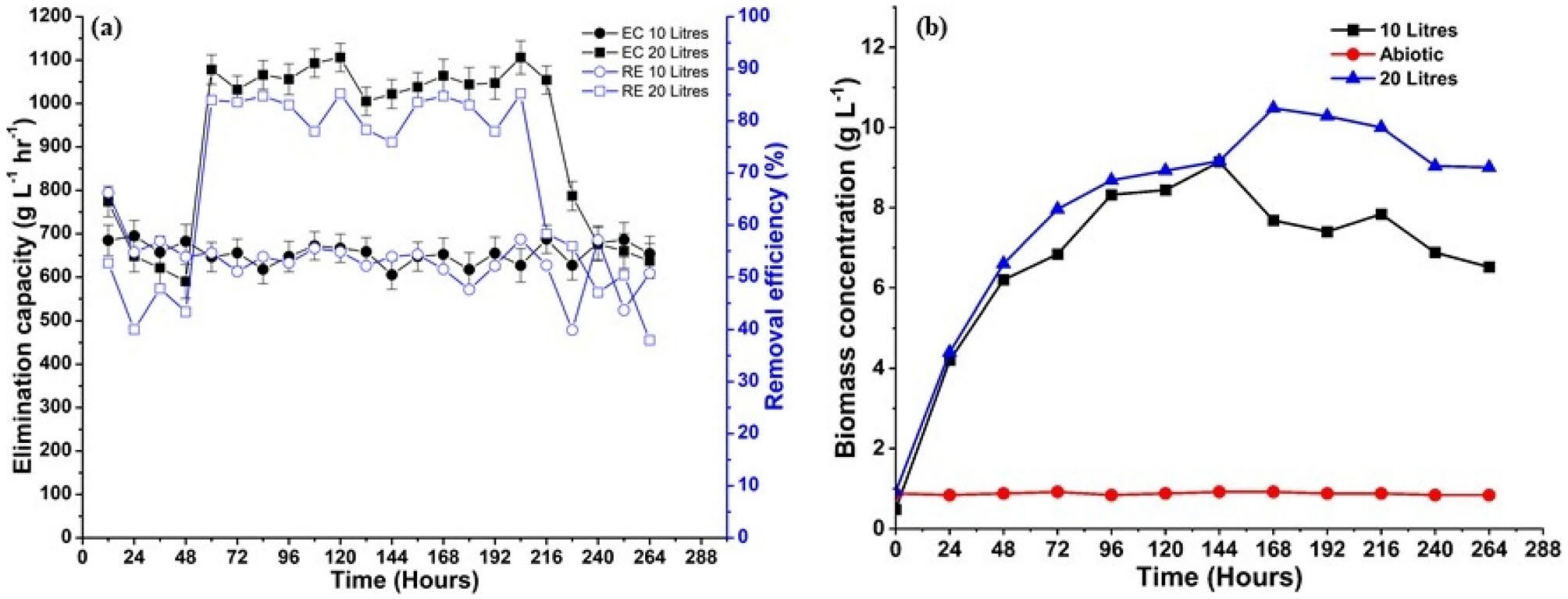
(**a**) The removal efficiency and elimination capacity of CO_2_ (g) bio-mitigation, and (**b**) the biomass concentration of 10 L and 20 L reactors run and abiotic test.

The main focus of the investigation was the bio-mitigation of CO_2_ (g) by thermophilic bacteria (*B. cereus* SSLMC2) in the bubble column reactor. Dissolved CO_2_ from the gaseous phase served as the carbon source and substrate for the organism’s growth. For both the 10 L and 20 L reactor systems, the CO_2_ (g) bio-mitigation efficiency was calculated by comparing the CO_2_ (g) concentration (%) at the reactor’s inlet and outlet. The bio-mitigation process’s elimination capacity and removal efficiency are shown in Fig. 3a. The highest removal efficiency, 56%, and 85%, was achieved in the 10 L and 20 L columns reactor, respectively. The removal efficiency of the two operational volumes varied due to the 20 L reactor’s series mode operation. The initial column breaks down and reduces a significant portion of the CO_2_ (g). A greater proportion of the leftover amount was used in the second column, leading to a higher rate of mitigation. The residence time of CO2 in the 20 L reactor system is nearly twice as long as in the 10 L reactor, potentially leading to increased mitigation. The elimination capacity is also determined based on the system's removal efficiency. The reactor with higher removal efficiency performs more and can be efficiently used for bio-mitigation. The elimination capacity values vary from 646.14 to 694.87 g L^−1^ h^−1^ and 590.0 to 1106.0 g L^−1^ h^−1^ due to the inlet and outlet concentration differences for the inlet load of 1200 g L^−1^ h^−1^ and 600 g L^−1^ h^−1^ for 10 L and 20 L reactor volumes, respectively. The present study indicates that; a larger volume reactor in a series mode operation is effective for increasing CO_2_ mitigation.

The initial high removal efficiency of 50% and 65% in both columns was due to the medium’s slow dissolution of CO_2_ (g) to achieve equilibrium during the lag phase (0–12 h). As a result, a large difference was obtained between the inlet and the outlet concentration that does not account for the utilization of CO_2_ by the bacterial species. Therefore, alterations in the rate of CO_2_ dissolution via changes in partial pressure of CO_2_, temperature, and interfacial area would impact the effectiveness of removing a percentage of CO_2_ during the lag phase (Gevantman [^37^]). Once the concentration of CO_2_ in the gaseous and liquid phases reached equilibrium, CO_2_ mitigation by *B. cereus* SSLMC2 started and simultaneously the growth phase started. Subsequently, the quantity of CO_2_ dissolved in the liquid phase equals the amount of CO_2_ consumed by bacteria. The decline phase of the bacteria begins at 169 h; the removal efficiency drops significantly from 55 to 37% and 85–55% due to the exhaustion of nutrient media and the death of bacteria.

The *B. cereus* SSLMC2 culture’s biomass concentration buildup is represented in Fig. 3b, which started with an exponential growth phase as the bacteria was already enriched with carbon source and nutrient media before inoculation. The mathematical correlation between the dry-weight biomass and OD values was obtained for the abiotic test, 10 and 20 L reactor run, represented in Supplementary Figs. S1–S3. The correlation obtained from the linear curve fit was utilized to calculate the biomass concentration of the reactor. The exponential phase was observed up to 120 h, where the bacteria multiplied throughout and attained the maximum growth. The bacteria utilized the maximum substrate and nutrients available in the media to grow in this phase. The exponential phase of bacterial development was the most productive in terms of biomass accumulation. The highest biomass concentration obtained was 9.14 g L^−1^ in the case of the 10 L reactor and 10.78 g L^−1^ in the case of the 20 L reactor system. The highest biomass productivity obtained for the 10 L and 20 L reactor systems were 0.061 g L^−1^ h^−1^ and 0.081 g L^−1^ h^−1^, respectively. The higher value of biomass concentration may be due to the fact that *Bacillus cereus* is an aerobic micro organism that can produce more energy by oxidizing inorganic compounds (CO_2_) due to the efficient role of O_2_ as an electron acceptor. It typically exhibits a higher growth rate because aerobic respiration is more efficient and faster at producing energy (ATP) for cellular functions. Aerobic cultivation typically leads to increased cell production per amount of substrate used due to the complete oxidation of the compounds^38^. The abiotic test was conducted in the reactor at the same operating conditions to test for the growth of *B. cereus* SSLMC2 in the absence of CO_2_ (Fig. 3b). The biomass concentration was deficient and remained almost constant along 0.8 g L^−1^. With the gaseous feed of only air, the bacteria showed no significant growth without a substrate, proving that they are solely dependent on CO_2_ (g) as a substrate for their growth.

The amount of CO_2_ used was directly related to the amount of CO_2_ available as a substrate and the amount of nutrients used by the bacteria^39^. The CO_2_ fixation rate calculated for the maximum biomass productivity was found to be 0.076 g L^−1^ h^−1^ and 0.1 g L^−1^ h^−1^ for the 10 and 20 L column reactor, respectively. Beyond 120 h, the culture's development began to decline exponentially due to the death of the bacteria, as the nutrients available in the media were limited. In this study, the reactor was operated in a semi-continuous mode in which media was supplied only at the start of the reactor, and the gaseous CO_2_ stream was fed continuously. The continuous supply of nutrients in equilibrium with nutrients’ input and output rates can lead to a longer time of bioreactor operation and increased biomass productivity. Finally, it reached a death phase at the end of 264 h, where the live bacteria may not be available to utilize the CO_2_ (g) as a carbon source. The dead bacterial biomass does not participate in the reactor system's mitigation or reaction process; hence, identifying the living cells is obligatory^40^.

### pH, DO, and salinity assessment of the reactor system

**Figure 4:**
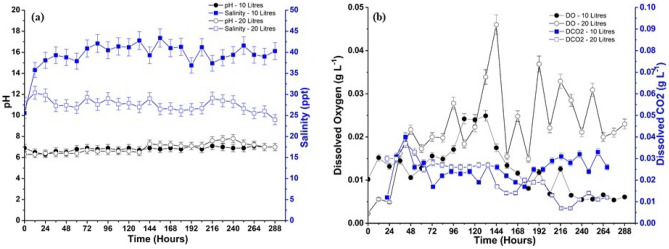
(**a**) pH and salinity variation and (**b**) DO and dissolved CO_2_ (g) variation in the 10 and 20 L bioreactor during the cultivation period.

The variation of pH and salinity throughout the cultivation period from inoculation to the last day is represented in Fig. 4a. The pH of the inoculum was 8.6 and dropped to 6.9 and 6.3 immediately after CO_2_ (g) induction to the 10 L and 20 L reactor, respectively. CO_2_ (g) combines with water to produce weak carbonic acid (H_2_CO_3_), which also contributes to the decrease in pH in the reactor. During the bio-mitigation process, the pH was observed between the range of 6–7.2 for the 10 L reactor and 6–7.8 for the 20 L reactor. There was an increase in the pH in the reactor due to continuous biomass formation and accumulation^23^. As the biomass growth started, the pH changed in the column due to the formation of enzymes and dissociation of dissolved CO_2_ (g). The pH in the reactor remained in the neutral range to enhance biomass accumulation. These factors increase and decrease the reactor's pH, making a balance to maintain the optimum pH for bacterial growth. The pH is more basic in the case of the 20 L reactor than the 10 L reactor during the stationary and death phase due to the higher biomass and inorganic carbon content accumulation rate. The threshold limit of salinity in the mixed bacterial culture obtained from the SSL is 70 ppt (parts per trillion)^41^. Therefore, maintaining salinity in the medium was crucial for the bacteria to adjust to the new environment in the reactor for optimal development. A high range of salinity would cause the rupture of the cells and premature death of the bacteria. The salinity in both reactor operation modes varied between 25 and 45 ppt, which is an optimum range for the working volume of the reactor^42^. The contribution of salinity in the medium was due to the addition of MSM. Both pH and salinity have similar trends; initially, they decreased, then increased and stabilized as the bacterial growth advanced. The media's alkalinity caused the increase and decrease in the pH values. Salinity change is assessed in relation to the original ions present in the medium (Na^+^, Cl^-^, K^+^). During the lag phase (0–12 h), bacteria’s metabolic activity causes the release of ions into the medium at a rate faster than they are absorbed, leading to a rise in salinity. While bacteria are growing, they absorb and release ions into the medium, which helps to maintain a stable salinity level^43^. Initially, there was abundant availability of nutrient media that sped the growth of the bacteria, and then equilibrium was attained due to the accumulation of biomass.

The entire bio-mitigation of CO_2_ (g) experiments was performed under aerobic conditions. Ample oxygen was provided to the reactor system through the air supplied and partially dissolved in the liquid medium. The variation in dissolved oxygen (O_2_) in the aqueous medium is represented in Fig. 4b. The concentration of DO in the media varied from 2 to 45 ppm, which was an aerobic environment for the bacteria to flourish^44^. Initially, the DO concentration was significantly lower in the medium, which started to increase after the supply of the gaseous mixture. The critical DO limit concentration for the bacteria in an aerobic environment is 9 ppm^44^. It is evident that the DO during the cultivation period was always above the critical DO concentration, as the highest achieved dissolved O_2_ value was 0.045 g L^−1^. The solubility of O_2_ in an aqueous NaCl solution is 0.041 g L^−1^ at 25 °C and 1 atm^45^. The brine solution formed due to the use of MSM and above atmospheric pressure in the reactor facilitated the dissolution of higher O_2_ in the media^46^. The bacteria utilize O_2_ to generate Adenosine triphosphate (ATP), the energy source for most cellular processes^19^. The level of dissolved O_2_ fluctuated throughout the cultivation period according to the consumption and conversion by the bacterial culture. pH, DO, and salinity were found to be influenced by *Bacillus* growth and media composition.

### Total inorganic and organic carbon calculation (TIC and TOC)

**Figure 5:**
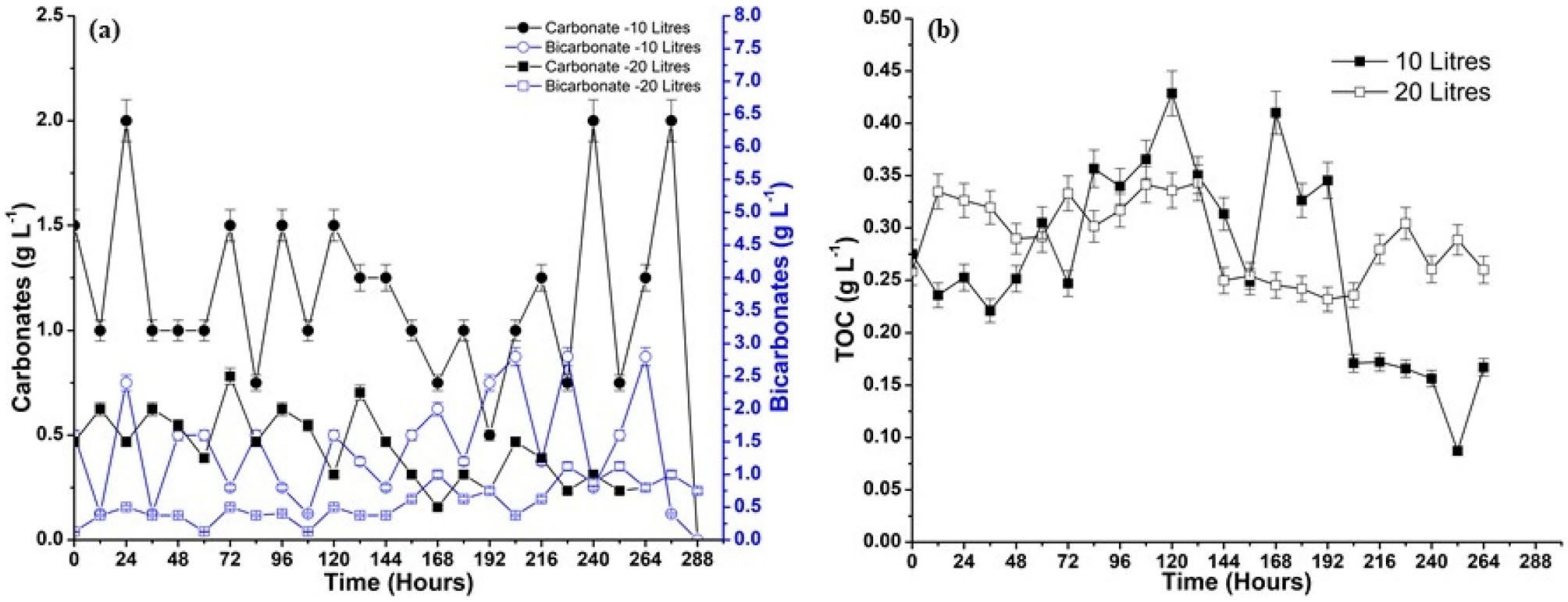
(**a**) The TIC and (**b**) TOC concentration in the reactor formed during cultivation.

Significant amounts of CO_2_ (g) were dissolved in the aqueous medium as the gaseous mixture flowed through it. Hence, the total carbon (TC) in the media in all forms was categorized into TIC and TOC. The TIC was further divided into three classifications: CO_3_^2−^, HCO_3_^−^, and dissolved CO_2_ (g). The concentration of dissolved CO_2_ (g) in the media is represented in Fig. 4 (b). Initially, the gas dissolution rate is higher in the media, which goes up to 0.040 g L^−1^. Later, the bacteria consume the CO_2_ (g) at an exponential rate for their growth, and the capacity for dissolution stabilizes due to the dissolved CO_2_ (g) accumulation. The dissolved CO_2_ (g) forms weak carbonic acid and is broken into carbonate and bicarbonate ions^47^. The formation of carbonate and bicarbonate ions in the media during the cultivation period is illustrated in Fig. 5a. The bacteria digest these inorganic components for their growth. The formation of carbonate and bicarbonate ions affects the media's pH. The media’s pH decreases as the bicarbonate alkalinity increases rather than the carbonate^48^. The formation of these TIC’s contributed to the mitigation of CO_2_ (g), and the remaining gas was let out as an outlet gas concentration.

The TOC is the carbon atoms that are covalently bonded in the organic molecules, i.e., the biomass. The empirical formula for the biomass was taken from the literature as C_6_H_17_O_7_N^38^. The TOC calculation and the carbon fraction obtained from the above formula give us information regarding the organic carbon content of the biomass^49^. The inorganic carbon content in the media is always greater than the organic content as the carbon attached to biomass is insufficient. The change in TOC with the cultivation period is represented in Fig. 5b. The organic content increased to 0.33 and 0.43 g L^−1^ for 10 L and 20 L, respectively, during the growth phase. Later, in the death phase, it decreased to 0.16 and 0.23 g L^−1^ for 10 L and 20 L, respectively. It also correlates with the dry-weight biomass data as it increases and decreases with the increase and decrease in dry-weight biomass. The organic content in the biomass is also responsible for lipid production. Large biomass production corresponds to more organic content, which results in the formation of by-products such as carboxylic acids. The elemental composition of the biomass, which has higher values of carbon, hydrogen, and oxygen, is responsible for good lipid yields. The increase or decrease in CO_2_ absorption by the bacterial cells also influences the yield of lipid production^36^.

### FT-IR analysis

**Figure 6:**
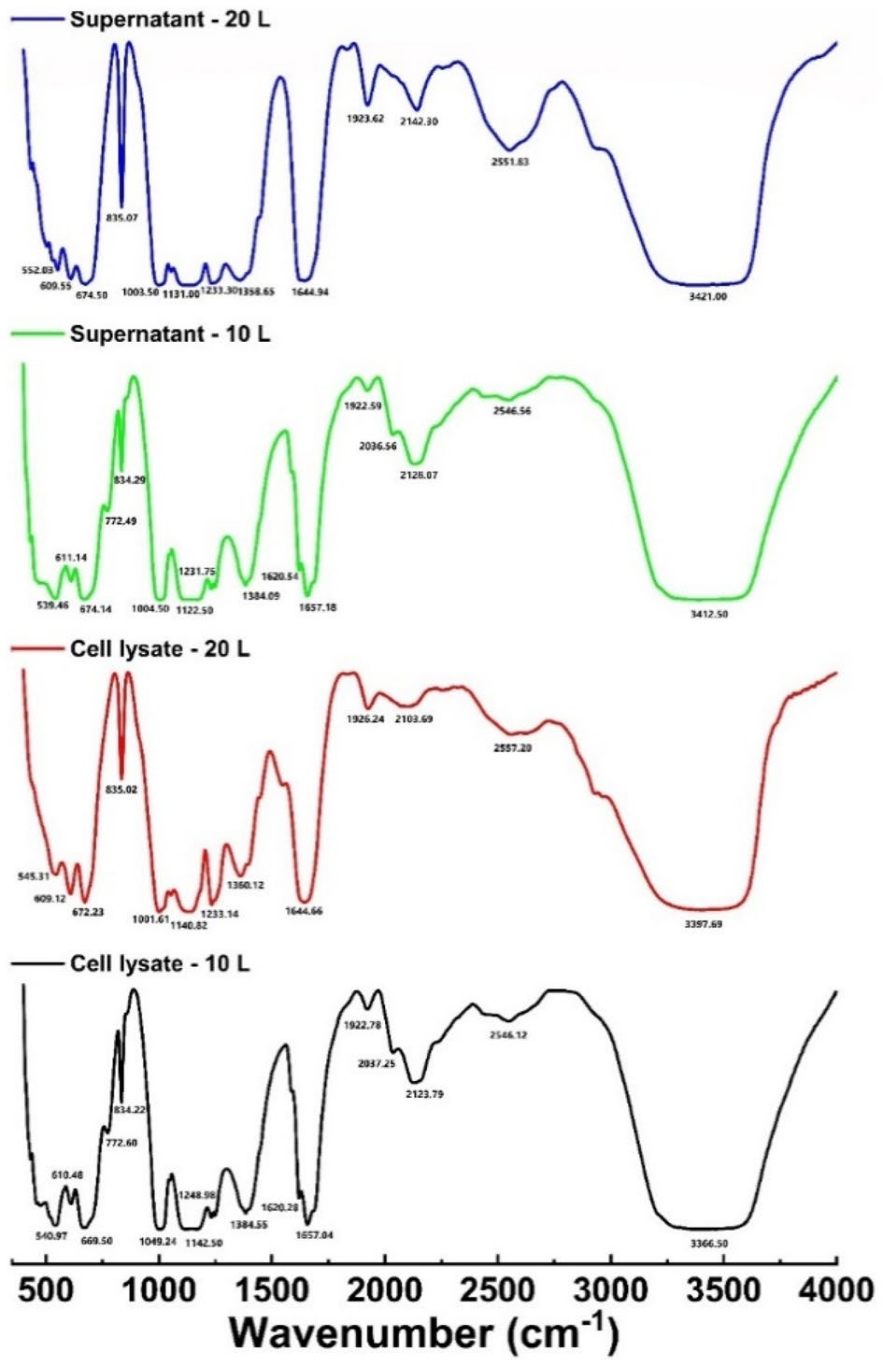
FT-IR spectra of the cell lysate and supernatant for 10 and 20 L bioreactor.

Cell lysate and cell-free supernatant samples collected during CO_2_ (g) bio-mitigation investigations from the 10 L and 20 L bioreactors were analyzed using FT-IR to identify important functional groups of bio-molecules such as proteins, lipids, carbohydrates, and nucleic acids and the obtained spectra are shown in Fig. 6. The characteristic absorption bands are highlighted, and the peak identification of the spectra was implemented as per the literature.

Cell lysate and cell-free supernatant spectra peaks ranged between 3500 and 400 cm^−1^. The major peaks were found in the region of 3500–3000 cm^−1^, 3000–2800 cm^−1^, 1700–1600 cm^−1^, 1500–1400 cm^−1^, 1300–1000 cm^−1^, and 800–600 cm^−1^. In FT-IR spectroscopy, the 3500–3000 cm^−1^ region is generally associated with the stretching vibrations of O–H bonds, which can be attributed to various functional groups, including alcohols (–OH), carboxylic acids (–COOH), phenols (–OH attached to an aromatic ring), and other hydroxyl-containing compounds^50^. The exact position and intensity of the peak can vary depending on the specific compound and its environment. The region between 3000 and 2800 cm^−1^ in the spectrum is associated with the stretching vibrations of C–H bonds, specifically the -CH groups of aliphatic hydrocarbons. Peaks observed in regions 1900–2200 cm^−1^ may be attributable to compounds present in the bacterial biomass, such as lipids and proteins. The 20 L reactor samples exhibit a notable augmentation in the peak in this particular region. This can be attributed to the substantial biomass generated throughout the cultivation process, which led to a greater accumulation of lipids and proteins than the 10 L reactor biomass^36^. In the case of cell-bound fatty acids, the peak in the 1700–1600 cm^−1^ region corresponds to the stretching vibration of the carbonyl group in the fatty acid molecules^51^. On the other hand, in proteins, the amide I band is located in the same region, and it arises from the stretching vibration of the C=O bond in the peptide backbone^52^. The peak at 2200 cm^−1^ is the absorption band of C≡C. Carboxylic acids can display a peak in the 1500–1400 cm^−1^ region due to the bending motion of the hydroxyl group. The carbonate ions can produce a peak in the vicinity of 1500–1400 cm^−1^^50^. In the 800–600 cm^−1^ region, one commonly observed peak corresponds to the bending vibrations of C-H bonds in long-chain alkanes. This peak is often referred to as the “C-H rock.” The presence of this peak suggests the presence of aliphatic hydrocarbons, such as long-chain alkanes^50^. Therefore, various functional groups associated with polysaccharides and carboxylic acids were defined across the whole FT-IR spectra of both cell lysate and supernatant for both reactor volumes.

### GC–MS analysis

After finding the functional groups through FT-IR analysis, the formed carboxylic acids, fatty alcohols, and hydrocarbons were identified through the GC–MS analysis. The analysis was conducted for the extracellular (EC) (cell-free supernatant) and the intracellular (IC) (cell lysate) products obtained during bio-mitigation. The identification in both cases exhibited a substantial amount of fatty alcohols, esters, aldehydes, carboxylic acids, and hydrocarbons with match quality with the mass spectra library ranging from 100 to 30% mentioned in Table 1. The metabolites identified in the extracellular and intracellular samples in the 10 and 20 L reactor volumes are represented in Supplementary Tables S1–S4.

**Table 1:** Metabolites (%) obtained from cell lysate and supernatant of 10 and 20 L bioreactor.

Reactor volume	Cell lysate			Supernatant		
	Carboxylic acid (%)	Fatty alcohol (%)	Hydrocarbons (%)	Carboxylic acid (%)	Fatty alcohol (%)	Hydrocarbons (%)
10 L	45.15	2.08	33.61	1.92	2.71	32.01
20 L	17.39	19.41	41.89	6.58	6.29	70.52

In the extracellular metabolite identification of the 10 and 20 L reactor run, the samples were identified for many compounds in significant amounts. The sample comprised of 2.71 and 6.29% of fatty alcohols, 32.01 and 70.52% of hydrocarbons, and 1.92 and 6.58% of carboxylic acids in the 10 and 20 L, respectively. The fatty alcohols found to span a carbon chain length spectrum from C_7_ to C_24,_ such as 1-decanol, methyl dodecanol, 1-hexacosanol, 1-tetradecanol, 1,2-propanediol, etc. At the same time, the carboxylic acid constituted of phthalic, benzene-propanoic, carbonic, succinic, trichloroacetic, 1–2 benzene dicarboxylic, diethylmalonic, pentanoic, oleic, erucic acid etc. The hydrocarbons ranged from carbon chain lengths of C_6_ to C_54,_ such as undecane, dodecane, tetradecane, heptadecane, pentadecane, hexadecane, nonadecane, tetrapentacontane, eicosane etc. The total carbon content of EC samples in the 10 and 20 L volumes was approximately 14.31 and 32.20 g, respectively.

A similar identification was made for the intracellular samples from the reactor, which produced compounds in greater quantity than the extracellular samples. The intracellular sample comprised 2.08 and 19.41% of fatty alcohols, 45.15 and 17.39% of carboxylic acids, and 33.61 and 41.89% of hydrocarbons in the 10 and 20 L reactor volumes, respectively. The major carboxylic acids were benzoic, 1,2 benzene dicarboxylic, benzene-propanoic, succinic, diglycolic, octadecanoic, hexadecanoic, 2-butene-dioic acids, etc. The fatty alcohols have carbon chain lengths ranging from C_5_ to C_21_ and hydrocarbons ranging from C_12_ to C_20_. The hydrocarbons were tetradecane, eicosane, pentadecane, undecane, tridecane, hexadecane, cyclohexane etc. In comparison, the fatty alcohols comprised of 1-decanol, 1-heptanol, 9-tetradecen-1-ol, 1-hexacosanol, 1-tetradecanol, 6-dodecanol etc. The carbon content of IC samples calculated in 10 and 20 L volumes was 17.42 and 52.70 g, respectively.

The mass of more compounds present in the intracellular sample than in the extracellular quantified that more reaction, digestion of CO_2,_ and production of by-products occur within the cell than in the surrounding of the cells. The FT-IR study confirms the functional group of all metabolites, and GC–MS emphasizes the presence of the approved compounds. In the GC–MS analysis, only the products with a substantial percentage of peak area were reported, and their mass was examined. The identified fatty alcohols, carboxylic acids, and hydrocarbons have many commercial uses in the pharmaceutical, cosmetic, food, and bio-fuel sectors^53,54^. Hence, it proves that the bio-mitigation of CO_2_ efficiently reduces flue gas and simultaneously produces many valuable products that can be used commercially.

### Carbon balance

The carbon balance equals the sum of the initial mass of carbon introduced into the system minus the final mass of carbon extracted from the system. The change in CO_2_ (g) concentration between the system's inlet and output was used to calculate the amount of carbon consumed. The total carbon balance of the entire system for both the volumes of the reactor is illustrated in Table 2.

**Table 2:** Total carbon balance of the inlet and outlet of the system.

Source of carbon (C) in the system	Mass of carbon (g) in 10 L column run	% Utilization	Mass of carbon (g) in 20 L column run	% Utilization
Total carbon supplied to the system as input through CO_2_ (g)	1696.13		1696.13	
Carbon withdrawn in the gaseous phase as CO_2_ (g) at the outlet of the system	774.36	45.65	509.40	30
Carbon present in the form of dissolved CO_2_ (g) in the reactor	3.96	0.23	62.40	3.68
Carbon present in the form of carbonates (CO_3_^2-^)	240	14.15	90	5.30
Carbon present in the form of bicarbonates (HCO_3_^-^)	105	6.19	90	5.30
Carbon assimilated in the form of biomass	523.22	30.85	835.20	49.24
Total carbon mitigated in the system	1646.54	97.07	1587	93.57
The difference in the mass of carbon in the inlet and outlet	49.59	2.92	109.13	6.43

The only carbon source provided to the entire system is the CO_2_ (g) mixture. Hence, the total mass of carbon (C) supplied in gaseous form was 1696.13 g in both the volumes of reactor operation. The mass of carbon withdrawn from the reactor in gaseous form was estimated as 774.36 g and 509.40 g in the 10 and 20 L reactor columns, respectively, calculated by the difference between the inlet and outlet concentration of the CO_2_ (g). The carbon in the form of dissolved CO_2_ (g) in the aqueous media was measured as 3.96 g and 62.20 g in 10 L and 20 L reactor columns, respectively. The carbon in the form of carbonates was 240 g and 90 g, and the bicarbonates were 105 g and 90 g for 10 L and 20 L reactors, respectively. The carbon assimilated as organic carbon in the form of biomass was found to be 523.22 g and 835.20 g for the 10 L and 20 L reactors, respectively. Therefore, the total CO_2_ (g) present in the output streams of bioreactors in all forms was 1646.54 g and 1587 g for the 10 L and 20 L reactors, respectively. The carbon mass difference between the inlet and outlet was 49.59 g and 109.13 g for 10 L and 20 L reactor systems, respectively, due to the extracellular product formation confirmed by the FT-IR and GC–MS analysis of cell-free supernatant. In both cases, the mass of carbon in the biomass is accounted for to a major extent in the GC–MS analysis, as some carbon present in the biomass may also be due to the product formation in the cell. Some mass of carbon in the products in the extracellular sample could not be quantified, contributing to the carbon mass difference. The estimation of the products produced in the system shown in the GC–MS section is the preliminary calculation. The downstreaming process of the samples can provide more insight into the products formed and confirm the exact quantity of the products produced in the extracellular samples as well.

### Techno-economic assessment for CO_2_ (g) bio-mitigation process

A commercial plant has several extra expenses besides operating and installed equipment expenditures. Several factors can be used to establish a direct correlation between most of these expenses. Table 3 concisely summarizes the techno-economic analysis of the total CO_2_ (g) bio-mitigation process based on the simplified cost model^55^.

**Table 3:** Breakdown of total capital investment, fixed capital investment, and working capital for the process.

Expenditure		Cost in $/reactor run		Cost in $/g of carbon treated	
		10 L	20 L	10 L	20 L
Working capital					
Raw materials (MSM)	US$	0.54	0.97	0.00033	0.00062
Energy consumption	US$	18.5	18.5	0.011	0.012
Workforce	US$	23.87	23.87	0.014	0.015
Total	US$	42.91	43.34	0.025	0.028
TCI (Total capital investment)	6.67 (Working capital)				
	US$	286.21	289.08	0.167	0.187
FCI (Fixed capital investment)	TCI—Working capital				
	US$	243.30	245.74	0.142	0.159
Direct cost	Onsite costs or Inside battery limits				
Purchased equipment (PE) (30% of FCI)	US$	72.99	73.72	0.043	0.048
Installation (40% of PE)	US$	29.20	29.49	0.017	0.019
Instrumentation and control (18% of PE)	US$	13.14	13.27	0.008	0.009
Electrical equipment (14% of PE)	US$	10.22	10.32	0.006	0.007
	Offsite costs or Outside battery limits				
	US$	0	0	0	0
Indirect Costs					
Engineering and supervision (12.5% of FCI)	US$	30.41	30.72	0.018	0.020
Contingency (12.5% of FCI)	US$	30.41	30.72	0.018	0.020
Start-up costs	9% of FCI				
	US$	21.90	22.12	0.015	0.017

The initial steps involve determining the necessary equipment sizes and utility loads (working capital) of the process, computed per reactor run of 11 days and per gram of carbon-treated basis. Based on the working capital, the cost models were utilized to estimate the operating and other expenses^55^. A breakdown of the overall working capital includes costs of MSM, commercial chemicals procured from the IndiaMart platform, energy usage for air compressor, pump, reactor central unit, and other electronics (electricity) (INR 8/unit for commercial use at BITS Pilani), and personnel compensation (salaries/labor charges). The detailed cost-wise break of each element of the working capital is described in the Supplementary Table S5. Based on the experimental run, the total working capital was projected to be $42.91 per reactor run, $0.025 per gram of carbon treated for a 10 L reactor, $43.34 per reactor run, and $0.028 per gram of carbon treated for a 20 L reactor run. A total of $21.90 and $22.12 for every reactor run (10 L and 20 L, respectively) and $0.015 and $0.017 per gram of carbon treated (10 L and 20 L, respectively) were calculated to represent the start-up costs allocated to equipment modification as part of the capital expenditure.

The fixed capital investment is the sum of the direct and indirect costs. Expenses incurred at a particular site, as opposed to those incurred elsewhere, are known as “direct expenses,” they consist of things like the price of materials and labour used to set up the necessary machinery and wiring (inside battery limits). The facilities required for the process but constructed in a distant place are known as “offsite costs” (outside battery limits). The calculated onsite costs for this procedure are $125.55 for a 10 L reactor run, $126.80 for a 20 L reactor run, $0.074 and $0.083 per gram of carbon treated. Indirect expenses include things like engineering, monitoring, and slack budgeting that aren’t accounted for in the planning stages. The calculated indirect costs for the procedure are $60.82 for 10 and 20 L reactor runs and $0.038 per gram of carbon treated. So, the estimated fixed capital investment for the process was $243.30 per reactor run for 10L, $245.74 for 20L, and $0.142 and $0.159 per gram of carbon treated, respectively. In the end, it was determined that the overall capital investment for the process, including both working capital and fixed capital investment, was projected to be $286.21 and $289.08 per reactor run for 10L and 20L, and $0.167 and $0.187 per gram of carbon treated, respectively. The total capital investment for this method represents the money spent on CO_2_ (g) bio-mitigation. In upscaling it from 10 to 20 L, we incurred a rise of 0.99% in the TCI per reactor run and a rise of 12% in TCI per gram of carbon treated. Hence, the techno-economic analysis proves that the process can be commercialized and scaled up to the industrial level cost-effectively.

### Mechanism of bio-mitigation of *Bacillus cereus* and comparison

**Figure 7:**
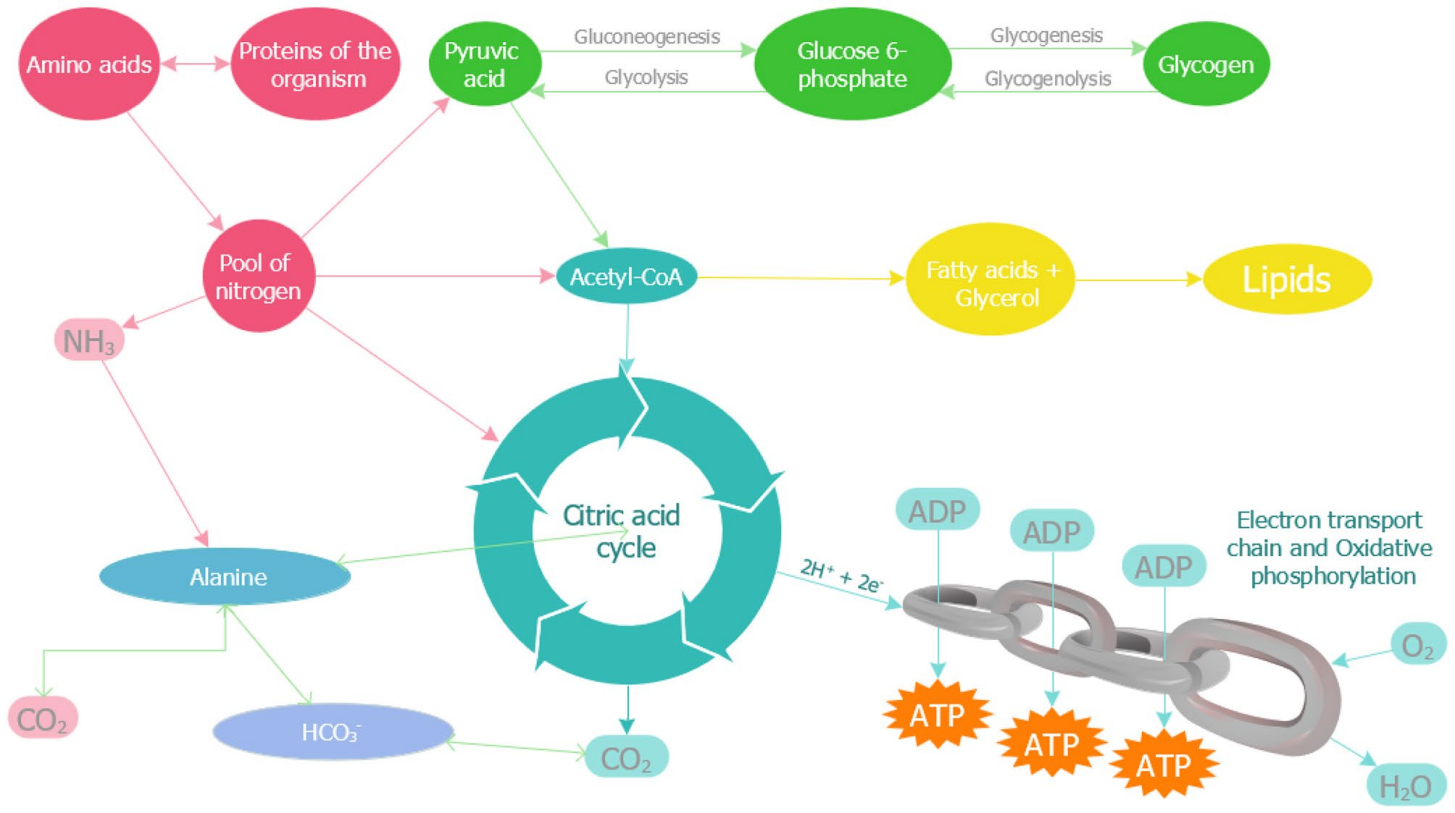
The suggested metabolic pathway of *B. cereus* SSLMC2 for bio-mitigation of CO_2_ (g).

The mitigation mechanism of CO_2_ (g) depends upon the species of the microorganism. The identified species *B. cereus* SSLMC2 carries out the metabolic process inside the cell to convert the CO_2_ compounds and produce value-added products^56,57^. A proposed metabolic pathway of *B. cereus* SSLMC2 is shown in Fig. 7.

For microorganisms to extract energy from their surroundings, they must undergo a series of oxidation and reduction reactions (ATP and NADH). Aside from acting as catalysts, microbes also serve as the end product of the reaction. Microorganisms are responsible for most metabolic reactions, require the energy they generate for cell synthesis, and continuously sustain cellular function. All processes use organic matter apart from photosynthetic organisms to obtain electron donors. Chemolithotrophic prokaryotes utilize electron transport via reduced inorganic compounds for energy metabolism. Diatomic or molecular oxygen (O_2_) is the electron acceptor in aerobic circumstances. Some prokaryotes can thrive in an anaerobic and aerobic environment using nitrates, sulphate, and CO_2_ as electron acceptors. The organic matter serves as both an electron acceptor and donor during fermentation processes. The preference for choosing an electron acceptor is oxygen, nitrate, sulphate, and carbon dioxide. To generate energy, the electron donor transfers electrons to the electron acceptor. Aerobic organisms must send a few electrons from their source to oxygen to get the energy required to synthesize a given amount of biomass. Consequently, aerobic bacteria are more efficient than their anaerobic relatives^38^.

In this study, *B. cereus* SSLMC2 was enriched in an aerobic environment; hence, the preferential electron acceptor for the organism was oxygen (O_2_). The inorganic half-reaction (Eq. 8) for the electron acceptor (R_a_), where the reduced-oxidized compounds are water-oxygen, is as follows:

8 $$\frac{1}{4} O_{2} + H^{ + } + e^{ - } \to \frac{1}{2} H_{2} O\;\;\;\Delta {\text{G}}^{{0^{\prime}}} = \, - {78}.{\text{72 kJ}}/{\text{e}}^{ - } \;{\text{eq}}$$

The energy source provided to the organism as an electron donor is the thiosulphate (S_2_O_3_^2−^) added through the MSM. The inorganic half-reaction (Eq. 2) for the electron donor (R_d_), where the reduced-oxidized compounds are thiosulphate-sulphate, is as follows:

9 $$\frac{1}{4} SO_{4}^{2 - } + \frac{5}{4}H^{ + } + e^{ - } \to \frac{1}{8} S_{2} O_{3}^{2 - } + \frac{5}{8} H_{2} O\;\;\Delta {\text{G}}^{{0^{\prime}}} = { 23}.{\text{58 kJ}}/{\text{e}}^{ - } \;{\text{eq}}$$

So, the energy reaction, R_e_, then becomes: Re (Eq. 10) = Ra (Eq. 8)–Rd (Eq. 9)

$$\begin{gathered} \frac{1}{4} O_{2} + H^{ + } + e^{ - } \to \frac{1}{2} H_{2} O\;\;\;\Delta {\text{G}}^{{0^{\prime}}} = \, - {78}.{\text{72 kJ}}/{\text{e}}^{ - } \;{\text{eq}} \hfill \\ - \hfill \\ \frac{1}{8} S_{2} O_{3}^{2 - } + \frac{5}{8} H_{2} O \to \frac{1}{4} SO_{4}^{2 - } + \frac{5}{4}H^{ + } + e^{ - } \;\;\;\Delta {\text{G}}^{{0^{\prime}}} = { 23}.{\text{58 kJ}}/{\text{e}}^{ - } \;{\text{eq}} \hfill \\ \end{gathered}$$

10 $$\frac{1}{8} S_{2} O_{3}^{2 - } + \frac{1}{8} H_{2} O + \frac{1}{4} O_{2} \to \frac{1}{4} SO_{4}^{2 - } + \frac{1}{4}H^{ + } \;\;\;\Delta {\text{G}}^{{0^{\prime}}} = \, - {1}0{2}.{3}0{\text{ kJ}}/{\text{e}}^{ - } \;{\text{eq}}$$

The total energy achieved by transferring electrons from donor to acceptor is − 102.30 kJ/e^−^ eq. Gibb’s free energy for the energy reaction is negative, which denotes that the reaction is spontaneous at a particular temperature, and the reaction favours the formation of products.

When organic compounds undergo half-reactions, CO_2_, CO_3_^2−^, or HCO_3_^−^ is always the oxidized form. The organic half-reaction (Eq. 11) for cell synthesis (R_c_), where CO_2_ and HCO_3_^−^ are the carbon source and ammonia (NH_4_^+^) is the nitrogen source, is as follows:

11 $$\frac{1}{5} CO_{2} + \frac{1}{20} NH_{4}^{ + } + \frac{1}{20} HCO_{3}^{ - } + H^{ + } + e^{ - } \to \frac{1}{20} C_{5} H_{7} O_{2} N + \frac{9}{20} H_{2} O$$

So, the synthesis reaction, R_s_, then becomes: R_s_ (Eq. 12) = R_c_ (Eq. 11)–R_d_ (Eq. 9)

$$\begin{gathered} \frac{1}{5} CO_{2} + \frac{1}{20} NH_{4}^{ + } + \frac{1}{20} HCO_{3}^{ - } + H^{ + } + e^{ - } \to \frac{1}{20} C_{5} H_{7} O_{2} N + \frac{9}{20} H_{2} O \hfill \\ - \hfill \\ \frac{1}{8} S_{2} O_{3}^{2 - } + \frac{5}{8} H_{2} O \to \frac{1}{4} SO_{4}^{2 - } + \frac{5}{4}H^{ + } + e^{ - } \hfill \\ \end{gathered}$$

12 $$\frac{1}{5} CO_{2} + \frac{1}{20} NH_{4}^{ + } + \frac{1}{20} HCO_{3}^{ - } + \frac{1}{8} S_{2} O_{3}^{2 - } + \frac{7}{40} H_{2} O \to \frac{1}{20} C_{5} H_{7} O_{2} N + \frac{1}{4} SO_{4}^{2 - } + \frac{1}{4}H^{ + }$$

In a bioreactor operating at a neutral pH, the nitrogen source is always assumed to be NH_4_^+^, as this species dominates in the aqueous solutions at a pH below 9.3. NH_4_^+^ acts as the nitrogen source for forming different proteins and nucleic acids. The protein and carbohydrate metabolism in the bacterial cell modulates the mitigation process along with the excretion of by-products in the form of fatty alcohols. The source of NH_4_^+^ was provided in the MSM by adding NH_4_Cl. When NH_4_^+^ is released following the oxidation of an organic compound, a negatively charged species must also be formed to balance the charge. Hence, the dominant species generally formed with organic oxidation of CO_2_ at neutral pH is HCO_3_^−^. The alanine (Eq. 13) and pyruvate (Eq. 14) metabolism involving CO_2_, NH4^+^, and HCO_3_^-^ depicts the metabolic changes taking place in the cell. $\frac{1}{12}$ moles of alanine oxidize to produce $\frac{1}{12}$ moles each of NH_4_^+^ and HCO_3_^-^. Similarly, $\frac{1}{10}$ moles of pyruvates oxidize to produce $\frac{1}{10}$ moles of HCO_3_^−^^38^.

13 $$\frac{1}{6} CO_{2} + \frac{1}{12} NH_{4}^{ + } + \frac{1}{12} HCO_{3}^{ - } + \frac{11}{{12}}H^{ + } + e^{ - } \to \frac{1}{12} CH_{3} CHNH_{2} COO_{ }^{ - } + \frac{5}{12} H_{2} O\;\;\;\Delta {\text{G}}^{{0^{\prime}}} = { 31}.{\text{37 kJ}}/{\text{e}}^{ - } {\text{eq}}$$

14 $$\frac{1}{5} CO_{2} + \frac{1}{10} HCO_{3}^{ - } + H^{ + } + e^{ - } \to \frac{1}{10} CH_{3} COCOO_{ }^{ - } + \frac{2}{5} H_{2} O\;\;\;\Delta {\text{G}}^{{0^{\prime}}} = { 35}.0{\text{9 kJ}}/{\text{e}}^{ - } {\text{eq}}$$

ATP and nicotinamide adenine dinucleotide (NAD) are two energy molecules synthesized from the energy produced during these processes (NADH). The tricarboxylic acid (TCA) cycle provides cells with their principal energy source and plays a pivotal role in aerobic metabolism. During pyruvate oxidation, acetyl coenzyme A (acetyl CoA) is generated, and this molecule's chemical energy is then used to decrease NADH. Hence, the TCA cycle produces the energy required by the bacterial cell to undergo the stress of the mitigation process. The TCA, alanine, and pyruvate metabolism processes enhance the cell’s mitigation process of CO_2_ (g)^58,59^. This proves the species' necessary metabolic pathways for CO_2_ mitigation are the TCA cycle, alanine metabolism, pyruvate metabolism, and energy metabolism, as depicted in Fig. 7.

Table 4 compares the reported literature for the removal efficiency and biomass production of CO_2_ (g) bio-mitigation using different culture conditions. All the studies mentioned were conducted in aerobic conditions or with air-containing O_2_. It is evident from the comparison made with previous literature on bio-mitigation of CO_2_ (g) that the bubble column bioreactor has noticeable results of removal efficiency of 56 and 85% with 10 L and 20 L volume of reactor columns compared to the bioreactors reported in the literature. The higher volume of the reactor could also be the reason for its higher reactor efficiency. The biomass production (g L^−1^) obtained was ~ 9 and ~ 10 times higher for 10 L and 20 L bioreactors, respectively, compared to the previous studies. The higher biomass production rate shows the feasibility of upgrading this process to an industrial scale. The *B. cereus* SSLMC2 used in the reactor may also be more effective for the CO_2_ (g) bio-mitigation than the other bacterial and microalgae species reported because of its mixotrophic nature, which makes it possible to use both inorganic and organic sources of carbon and also different energy sources. This species also survives at higher temperatures and harsh conditions; hence, it can withstand the reactor’s extreme salinity and temperature conditions to mitigate CO_2_.

**Table 4:** Comparison of the present study with reported literature for CO_2_ (g) bio-mitigation under aerobic conditions.

Reactor system	Inlet concentration in gas (v/v%)	Cultivation days (hours)	Removal efficiency (%)	Biomass production (g L^−1^)	Species	References
Hollow fiber bioreactor	2–1	36	85	–	*Spirulina platensis*	Kumar et al*.*^60^
Batch BOD incubator shaker	13	120	84.6	0.386	*Bacillus cereus*	Mishra et al.^36^
Chemostat	5	80	85	–	*Serratia* sp.	Bharti et al.^15^
CSTR	5	60	75	–	*Bacillus* sp.	Sundaram & Thakur^16^
Flask batch	15	144	98	1.12	*Halomonas stevensii*	Mishra et al.^23^
Photobioreactor	20	480	87	0.012	*Chlorella vulgaris*	Almomani^61^
Erlenmeyer flask-type photo-bioreactors	10	168	80	0.56	*Chlorella fusca*	Duarte et al.^62^
Photo-bioreactor tank	0.04, 10, 20 and 40	240	0.03, 0.34, 0.22 and 0.24 g L^−1^ d^−1^	0.32, 1.16, 0.82, 0.89	*Leptolyngbya* sp. KC45	Pekkoh et al*.*^63^
Photo-bioreactor	10	192	0.48 g L^−1^ d^−1^	0.218 g L^−1^ d^−1^	*Chlorella vulgaris* & *Pseudomonas* sp.	Yu et al*.*^64^
Bubble column bio-reactor (10 and 20 L)	10	264	56 and 85	9.14 and 10.78	*B. cereus* SSLMC2	Present study

## Conclusions

Experiments for bio-mitigation of a 10% CO_2_ (g) gas mixture using *B. cereus* SSLMC2 were carried out in an indigenous pilot-scale bubble column reactor of 10 L and 20 L capacity. *B. cereus* SSLMC2’*s* biomass increased directly with CO_2_ (g) reduction, proving the species’ ability to thrive in environments with elevated gas levels. Large-scale operations could benefit from using a bubble column bioreactor, as evidenced by its much greater biomass yield (9.14 g L^−1^ for 10 L and 10.78 g L^−1^ for 20 L) and removal efficiency (56% for 10 L and 85% for 20 L) compared to reactors reported in the literature. The practical implementation of this research involves the development of an environmentally friendly and sustainable industrial procedure to reduce and control CO_2_ emissions in industrial settings. It can be applied directly at the point of emission in any industry. This work aims to mitigate the atmospheric pollution contributing to global warming and climate change. The bacteria required for cultivation can be acquired from any nearby industrial source. The generated biomass has the potential to yield biodiesel, bio-ethanol, biodegradable plastics, and organic acids, all of which find applications in the paint, pharmaceutical, and cosmetic sectors. The water produced after biomass extraction can be reused and repurposed for bacterial cultivation. The water can be discharged into the soil as it contains nutrients enhancing soil health, plants, and organisms. The design of bioreactors for bacterial mitigation is straightforward as it does not necessitate any specialized equipment, thus making it cost-effective. This research reveals new possibilities for using bubble column bioreactors and thermophilic bacteria on an industrial scale for CO_2_ (g) bio-mitigation.

### Supplementary Information

Supplementary Information.

## Data Availability

The datasets generated during the current study are available from the corresponding author upon reasonable request.
