# Supplementary material for: Reforming CO2 bio-mitigation utilizing Bacillus cereus from hypersaline realms in pilot-scale bubble column bioreactor
Source: Sci Rep. 2024 Mar 16;14:6354. doi: 10.1038/s41598-024-56965-8 (PMC10943127; doi:10.1038/s41598-024-56965-8)
Supplement: Supplementary file 1 — Supplementary Information. [file 41598_2024_56965_MOESM1_ESM.pdf]

Reforming CO_2 bio-mitigation utilizing *Bacillus cereus* from hypersaline realms in pilot-scale bubble column bioreactor

Rachael J Barla[1], Smita Raghuvanshi[1*], Suresh Gupta[1]

[1]Department of Chemical Engineering, Birla Institute of Technology and Science (BITS), Pilani- 333031, Rajasthan, India

[*]Corresponding Author: Address: Faculty Division-1, Department of Chemical Engineering, BITS PILANI, Pilani – 333031, Rajasthan, India, Tel.: +91-1596-515638 (O), Email: smita@pilani.bits-pilani.ac.in

Supplementary materials

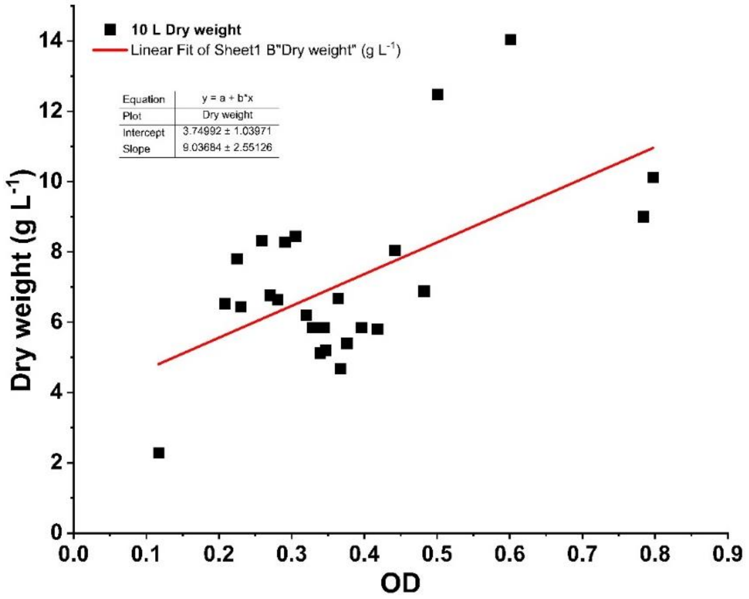

Fig. S1. The OD-dry weight biomass curve for the 10 L bioreactor

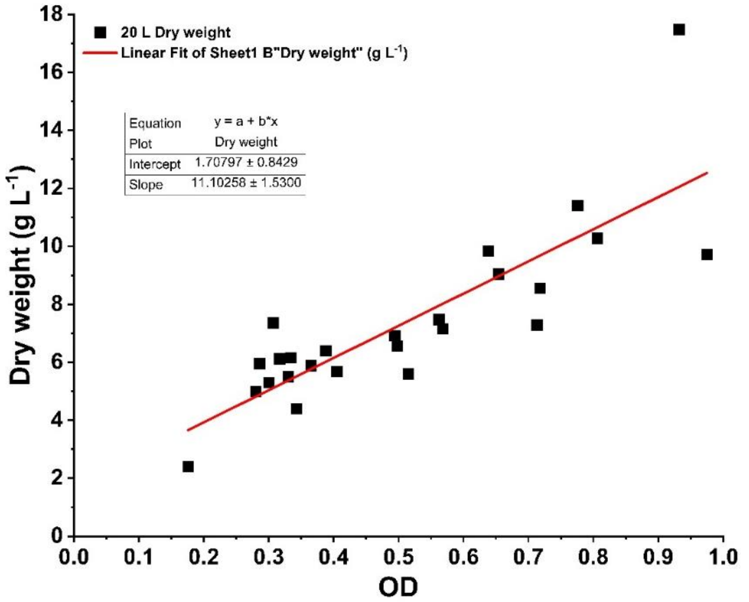

Fig. S2. The OD-dry weight biomass curve for the 20 L bioreactor

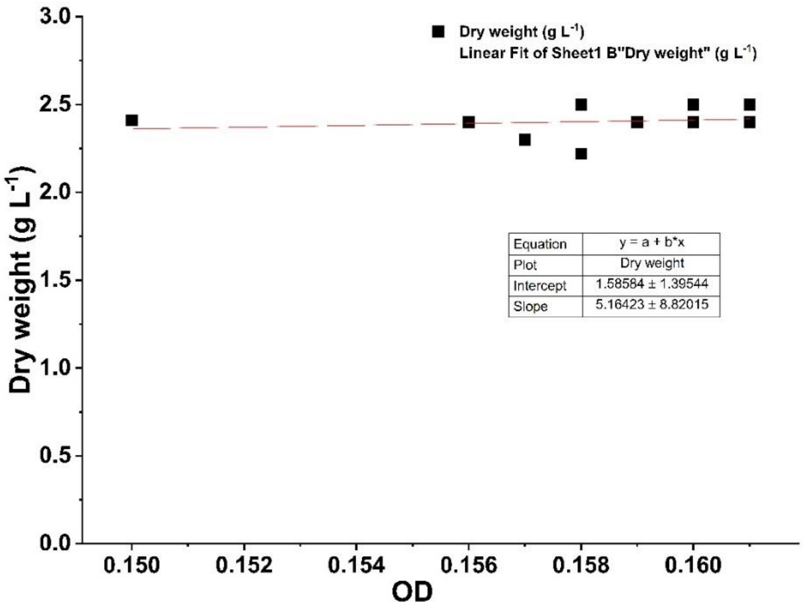

Fig. S3. The OD-dry weight biomass curve for the abiotic test

Table S1 GC-MS analysis of cell pellet obtained from 10 L reactor volume

Run time	Compound	% Area	Match quality
53.064	9-Borabicyclo[3.3.1]nonane, 9-ethoxy-	0.06	100
10.279	Benzenemethanesulfonyl fluoride	5.47	99
20.232	1-Pentadecene	1.14	99
26.462	1,2-Benzenedicarboxylic acid, bis(2-methylpropyl) ester	42.82	99
28.544	Dibutyl phthalate	13.36	99
17.593	Dodecane, 2,6,11-trimethyl-	0.67	98
22.794	Eicosane	2.08	98
23.069	1,2-Benzenedicarboxylic acid, butyl methyl ester	0.67	98
15.102	1-Tridecene	0.44	97
15.317	Tetradecane	0.34	97
20.105	Diethyl Phthalate	1.26	97
22.909	2,6,10-Trimethyltridecane	0.33	96
24.977	1-Heptadecene	0.57	96
27.983	Benzenepropanoic acid, 3,5-bis(1,1-dimethylethyl)-4-hydroxy-, methyl ester	0.43	95
18.228	2,4-Di-tert-butylphenol	0.51	94
11.95	Nonane, 5-(2-methylpropyl)-	0.17	93
20.417	Pentadecane	0.31	93
27.488	7,9-Di-tert-butyl-1-oxaspiro(4,5)deca-6,9-diene-2,8-dione	0.88	93
32.05	Hexacosane, 1-iodo-	0.34	93
23.456	1-Decanol, 2-hexyl-	0.14	92
41.282	Bis(2-ethylhexyl) phthalate	1.09	92
21.048	Undecane, 2-methyl-	0.08	90
17.482	Octadecane, 1-iodo-	0.12	89
50.86	Silane, dimethyl(2,2,2-trichloroethoxy)tridecyloxy-	0.17	89
7.427	Benzoic acid, methyl ester	0.57	88
18.316	1-Heptanol, 2,4-diethyl-	0.18	88
19.082	1,6-Dioxacyclododecane-7,12-dione	0.22	88
32.92	Triacontane, 1-iodo-	0.14	88
22.694	Decane, 4-ethyl-	0.15	87

36.321	Eicosyl isopropyl ether	0.56	87
53.5	d-Mannitol, 1,1'-O-1,16-hexadecanediylbis-	0.05	86
43.41	E-8-Methyl-9-tetradecen-1-ol acetate	0.25	82
21.556	Tridecane, 4,8-dimethyl-	0.07	81
16.95	Octadecane, 5-methyl-	0.13	79
22.125	Hexadecane, 2-methyl-	0.08	79
43.853	Succinic acid, 2,4,6-trichlorophenyl dec-4-en-1-yl ester	0.61	78
45.187	1,25-Dihydroxyvitamin D3, TMS derivative	0.39	63
52.805	Tricyclo[5.3.1.0(6,11)]undecane-11-carboxylic acid, 1,5,5-trimethyl-8-oxo-	0.05	60
44.474	Ambrial	0.58	52
46.205	1-Chloro-1-n-decyloxy-1-silacyclopentane	0.15	37
44.72	Glycine, N-(trifluoroacetyl)-, 1-methylbutyl ester	1.95	34

Table S2 GC-MS analysis of supernatant obtained from 10 L reactor volume

Run time	Compound	% Area	Match quality
23.14	1,2-Benzenedicarboxylic acid, butyl methyl ester	0.51	99
20.297	1-Pentadecene	0.7	99
10.378	Benzenemethanesulfonyl fluoride	12.9	99
5.628	Benzyl chloride	4.93	99
28.633	Dibutyl phthalate	5.98	99
25.049	E-15-Heptadecenal	0.41	99
18.814	Eicosane	1.63	99
15.162	1-Tridecene	0.26	98
18.291	2,4-Di-tert-butylphenol	0.26	98
28.065	Benzenepropanoic acid, 3,5-bis(1,1-dimethylethyl)-4-hydroxy-, methyl ester	0.24	98
12.011	Dodecane, 2,6,11-trimethyl-	0.32	98
17.543	Hexadecane	0.46	98
21.62	Pentadecane, 2,6,10-trimethyl-	0.08	98
15.38	Tetradecane	0.19	98
20.168	Diethyl Phthalate	0.47	97
29.48	1-Heptadecene	0.19	96
16.951	2,6,10-Trimethyltridecane	0.06	96
41.473	Bis(2-ethylhexyl) phthalate	0.41	96
23.829	Decane, 1-iodo-	0.05	96
33.012	Hexacosane, 1-iodo-	0.19	96
17.007	Undecane, 2,4-dimethyl-	0.09	96
23.527	1-Decanol, 2-hexyl-	0.21	94
25.211	Heneicosane	0.14	94
16.831	Pentadecane	0.33	94
27.57	Phthalic acid, 2,7-dimethyloct-7-en-5-yn-4-yl isobutyl ester	0.49	94
18.365	tert-Hexadecanethiol	0.07	94
26.124	Carbonic acid, hexadecyl prop-1-en-2-yl ester	0.09	93
21.868	Dodecane, 4-methyl-	0.05	93
20.672	Nonadecane	0.18	93
22.195	2-Bromotetradecane	0.06	92
15.778	Heptadecane	0.3	92
24.538	Octadecane, 2-methyl-	0.06	92
43.323	Trichloroacetic acid, tetradecyl ester	0.28	92
25.331	4-Methyldocosane	0.12	91

19.573	Heptadecane, 2,6,10,15-tetramethyl-	0.07	91
26.09	L-Lysine, N2,N6-bis(trifluoroacetyl)-5-[(trifluoroacetyl)oxy]-, butyl (ester)	0.04	91
19.751	Pentadecane, 3-methyl-	0.06	91
34.29	11-Methyldodecanol	0.05	90
27.84	Tetrapentacontane	0.46	90
10.016	Dodecane	0.13	89
37.354	Eicosane, 1-iodo-	0.16	88
5.032	Benzaldehyde	0.22	87
28.305	Carbonic acid, octadecyl vinyl ester	0.08	86
7.45	Undecane	0.13	84
35.926	2-Nonenal, 2-pentyl-	0.06	82
28.982	Tetradecane, 2,6,10-trimethyl-	0.1	80
21.929	Cyclohexane, undecyl-	0.05	79
5.2	Ethane, 1,1,2-trichloro-2-fluoro-	0.12	72
32.215	Succinic acid, 2-ethylhexyl 1,1,1-trifluoro-2-propyl ester	0.07	66
35.805	Diethylmalonic acid, 2-ethylphenyl 2-methylhex-3-yl ester	0.09	48
36.617	1,6-Nonadien-3-ol, 3,7-dimethyl-	0.12	33

Table S3 GC-MS analysis of cell lysate obtained from 20 L reactor volume

Run time	Compound	% Area	Match quality
25.24	1-Hexacosanol	9.87	100
13.71	1-Nonadecene	22.5	100
9.218	1-Tetradecanol	1.92	100
15.982	2-Propenoic acid, pentadecyl ester	2.05	100
13.549	Trichloroacetic acid, 2-octyl ester	0.45	100
16.646	1,2-Benzenedicarboxylic acid, butyl methyl ester	1.17	99
12.304	2,4-Di-tert-butylphenol	4.25	99
10.386	4-Fluoro-1-methyl-5-carboxylic acid, ethyl(ester)	0.53	99
14.206	6-Dodecanol acetate	0.36	99
20.995	Benzenepropanoic acid, 3,5-bis(1,1-dimethylethyl)-4-hydroxy-, methyl ester	0.34	99
30.977	Bis(2-ethylhexyl) phthalate	0.78	99
17.294	Cyclic octaatomic sulfur	3.15	99
21.425	Dibutyl phthalate	6.76	99
14.086	Diethyl Phthalate	0.46	99
25.311	Docosane	0.28	99
18.005	Eicosane	1.12	99
13.435	Erucic acid	0.86	99
13.835	Hexadecane	0.83	99
11.384	Hexathiane	2.95	99
46.794	Tris(2,4-di-tert-butylphenyl) phosphate	1.43	99
21.613	2-Hexadecene, 3,7,11,15-tetramethyl-, [R-[R*,R*-(E)]]-	0.57	98
12.55	Thiophene, 2-[(methylthio)ethynyl]-	0.88	98
11.499	Undec-10-ynoic acid, tetradecyl ester	1.19	98
28.251	[1,1'-Biphenyl]-2,3'-diol, 3,4',5,6'-tetrakis(1,1-dimethylethyl)-	0.15	97
5.025	6-Hydroxyhexahydrocyclopenta[b]furan-2-one	0.11	97
40.383	Heptacosyl heptafluorobutyrate	0.3	97
11.622	Heptadecane	0.26	97
20.591	Hexadecanoic acid, methyl ester	0.18	97
20.543	7,9-Di-tert-butyl-1-oxaspiro(4,5)deca-6,9-diene-2,8-dione	0.42	96

17.523	Benzyl Benzoate	0.56	96
18.167	Heneicosane, 11-(1-ethylpropyl)-	0.39	96
25.029	Octadecanoic acid	0.25	96
15.725	Phenol, 2,4-di-t-butyl-6-nitro-	0.58	96
35.58	Tetrapentacontane	0.21	96
17.756	Tridecane, 3-methylene-	0.28	96
10.213	Undec-10-ynoic acid, undecyl ester	0.83	96
19.591	1,2-Benzenedicarboxylic acid, bis(2-methylpropyl) ester	0.14	95
15.019	4-Methyldocosane	0.38	95
23.735	9-Octadecenoic acid, methyl ester, (E)-	0.14	95
18.648	Isopropyl myristate	0.43	95
15.515	Undec-10-ynoic acid, tridec-2-yn-1-yl ester	0.9	95
15.239	(R,Z)-2-Methyl-6-(4-methylcyclohexa-1,4-dien-1-yl)hept-2-en-1-ol	0.48	94
13.278	4-(para-Tolyl)-butyric acid	1.23	94
6.8	Cyclohexanepropanoic acid	2.5	94
8.417	Oleic Acid	0.38	94
28.526	Tetracosane	0.16	94
33.684	1,3-Benzenedicarboxylic acid, bis(2-ethylhexyl) ester	0.23	93
5.825	Benzenemethanesulfonyl fluoride	1.53	93
13.017	Oxirane, hexadecyl-	0.29	93
10.839	Silane, diethyldiheptyloxy-	0.34	93
12.713	1-Ethyl-4,4-dimethyl-cyclohex-2-en-1-ol	2.12	92
14.536	2-Methyl-Z-4-tetradecene	0.27	92
9.627	Cyclopentanecarboxylic acid, 1-methyl-3-(1-methylethyl)-, cis-	0.74	92
43.707	17-Pentatriacontene	0.19	91
8.243	Bicyclo[2.2.1]heptan-2-ol, 1-methyl-, acetate	0.26	91
11.046	Dimethyl phthalate	0.2	91
9.377	Tetradecane	1.52	90
8.326	1,4-Cyclohexanedicarboxylic acid	0.6	89
13.976	3-Cyclopentylpropionic acid, 3-tetradecyl ester	0.39	89
5.195	Nonane, 3-methyl-5-propyl-	0.3	89
12.861	6-Phenyl-2,4,4,6-tetramethyl-5,6-dihydro-4H-1,3-oxazine	0.69	88
11.788	Cyclohexanecarboxylic acid, 4-hexyl-	1.11	88
12.451	5-Mercapto-1H-[1,2,3]triazole-4-carboxylic acid cyclohexyl ester	0.57	87
10.967	2,5-Cyclohexadiene-1,4-dione, 2,6-bis(1,1-dimethylethyl)-	0.4	86
11.902	Carbonic acid, but-2-yn-1-yl octadecyl ester	0.15	85
13.923	trans-4-Pentylcyclohexanecarboxylic acid	0.3	85
17.371	Benzene, (1,1,4,6,6-pentamethylheptyl)-	0.29	83
14.954	Octanoic acid, cyclohexyl ester	0.16	82
16.056	Pentadecane, 2,6,10,14-tetramethyl-	0.26	82
11.283	Dihydronopol	0.77	81

Table S4 GC-MS analysis of supernatant obtained from 20 L reactor volume

Run time	Compound	% Area	Match quality
16.637	1,2-Benzenedicarboxylic acid, butyl methyl ester	3.49	100
13.684	1-Nonadecene	16.24	100
30.97	Bis(2-ethylhexyl) phthalate	1.42	100
11.33	Eicosane	3.368	100
15.384	Heneicosane	0.12	100
20.337	Tetrapentacontane	5.28	100
46.795	Tris(2,4-di-tert-butylphenyl) phosphate	11.84	100

25.216	1-Hexacosanol	1.85	99
9.221	1-Tetradecanol	2.06	99
12.292	2,4-Di-tert-butylphenol	1.69	99
20.99	Benzenepropanoic acid, 3,5-bis(1,1-dimethylethyl)-4-hydroxy-, methyl ester	0.87	99
21.413	Dibutyl phthalate	28.44	99
14.085	Diethyl Phthalate	0.95	99
7.211	Naphthalene, 1-methyl-	0.71	99
17.993	Pentatriacontane	0.45	99
9.379	Tetradecane	0.73	99
29.326	1,2-Propanediol, 3-benzyloxy-1,2-diacetyl-	0.29	98
10.758	2,6,10-Trimethyltridecane	0.72	97
23.731	9-Octadecenoic acid, methyl ester, (E)-	0.38	97
15.959	Dodecyl nonyl ether	0.73	97
18.883	Dotriacontane	0.52	97
17.317	Heptafluorobutyric acid, n-octadecyl ester	0.19	97
20.585	Hexadecanoic acid, methyl ester	0.71	97
25.135	Octacosyl trifluoroacetate	0.25	97
19.586	Phthalic acid, hex-3-yl isobutyl ester	0.26	97
12.516	Tetracosane	0.86	97
13.549	Tridecane, 3-methylene-	0.29	97
25.303	11-Methyltricosane	0.24	96
13.988	2-Hexadecene, 3,7,11,15-tetramethyl-, [R-[R*,R*-(E)]]-	2.39	96
14.674	Benzophenone	0.39	96
14.807	Carbonic acid, decyl undecyl ester	0.19	96
11.061	Dimethyl phthalate	0.22	96
6.754	Dodecane, 4,6-dimethyl-	0.23	96
18.162	Heneicosane, 11-(1-ethylpropyl)-	0.69	96
15.646	Heptadecane, 2-methyl-	0.36	96
11.626	Hexadecane	1.23	96
9.555	Naphthalene, 1,3-dimethyl-	0.3	96
5.025	9-octadecenoic acid, 2,2,2-trifluoroethyl ester	0.18	95
31.791	1,1,3,6-tetramethyl-2-(3,6,10,13,14-pentamethyl-3-ethyl-pentadecyl)cyclohexane	0.13	95
15.487	8-Octadecanone	0.24	95
14.867	Pentadecane, 2,6,10-trimethyl-	0.29	95
12.625	Thiophene, 2-[(methylthio)ethynyl]-	0.53	95
17.399	13-Methylheptacosane	0.14	93
23.523	Hexacontane	0.59	93
18.648	Isopropyl myristate	0.25	93
20.715	1,14-Dibromotetradecane	0.33	92
15.081	Hexadecane, 4-methyl-	0.23	92
16.733	Nonadecyl pentafluoropropionate	0.15	90
22.049	Cyclic octaatomic sulfur	0.29	89
12.801	2-Butenedioic acid (Z)-, dibutyl ester	0.13	88
18.97	2-Pentadecanone, 6,10,14-trimethyl-	0.25	88
20.531	7,9-Di-tert-butyl-1-oxaspiro(4,5)deca-6,9-diene-2,8-dione	0.24	88
14.951	Eicosane, 2-cyclohexyl-	0.27	88
16.306	5,5-Diethylpentadecane	0.37	87
23.833	1-Decanol, 2-hexyl-	0.17	86
21.288	2-Methylhexacosane	0.23	86

22.167	Cyclopropanecarboxylic acid, 1-hydroxy-, (2,6-di-t-butyl-4-methylphenyl) ester	0.18	86
20.49	Bis(tridecyl) phthalate	0.19	83
21.206	2,4-Dimethyl-3-pentanol acetate	0.23	77
5.124	Tridemorph	0.65	72

Table S5. Cost breakdown of the working capital for the techno-economic assessment.

Working capital expenditure	Commodity	Cost (US$)
MSM (cost per gram)	Nutrient broth	0.018
	Potassium Nitrate	0.0018
	Dipotassium hydrogen phosphate	0.0018
	Ammonium Chloride	0.059
	Sodium Thiosulphate	0.00057
	Sodium Chloride	0.0000061
Energy consumption @ 0.096 US$ /unit electricity	Air compressor (8 units/day)	8.45
	Pump (3 units/day)	3.17
	Reactor central unit (4.5 units/day)	4.77
	Flue gas analyzer (2 units/day)	2.11
Workforce (per day)	Labor charges	2.17

Figure and Table legends

Fig. S1. The OD-dry weight biomass curve for the 10 L bioreactor
Fig. S2. The OD-dry weight biomass curve for the 20 L bioreactor
Fig. S3. The OD-dry weight biomass curve for the abiotic test
Table S1 GC-MS analysis of cell pellet obtained from 10 L reactor volume
Table S2 GC-MS analysis of supernatant obtained from 10 L reactor volume
Table S3 GC-MS analysis of cell lysate obtained from 20 L reactor volume
Table S4 GC-MS analysis of supernatant obtained from 20 L reactor volume
Table S5. Cost breakdown of the working capital for the techno-economic assessment.
